# Using Custom Fiber Bragg Grating-Based Sensors to Monitor Artificial Landslides

**DOI:** 10.3390/s16091417

**Published:** 2016-09-02

**Authors:** Qinghua Zhang, Yuan Wang, Yangyang Sun, Lei Gao, Zhenglin Zhang, Wenyuan Zhang, Pengchong Zhao, Yin Yue

**Affiliations:** 1 College of Defense Engineering, PLA University of Science and Technology, Nanjing 210007, China; sqmha@126.com (Q.Z.); bryant8011@163.com (Y.S.); 54061@njnu.edu.cn (L.G.); plaust9110@163.com (Z.Z.); zwy282115810@163.com (W.Z.); blouson@163.com (P.Z.); 15251895292@163.com (Y.Y.); 2 State Key Laboratory of Disaster Prevention and Mitigation of Explosion and Impact, Nanjing 210007, China; 3 College of Mechanical Engineering, Nanjing University of Science and Technology, Nanjing 210094, China

**Keywords:** artificial landslide, FBG, sensors, monitor

## Abstract

Four custom fiber Bragg grating (FBG)-based sensors are developed to monitor an artificial landslide located in Nanjing, China. The sensors are composed of a rod and two FBGs. Based on the strength of the rods, two sensors are referred to as “hard sensors” (Sensor 1 and Sensor 2), the other two are referred to as “soft sensors” (Sensor 3 and Sensor 4). The two FBGs are fixed on each sensor rod at distances of 50 cm and 100 cm from the top of the rod (an upper FBG and a lower FBG). In the experiment presented in this paper, the sensors are installed on a slope on which an artificial landslide is generated through both machine-based and manual excavation. The fiber sensing system consists of the four custom FBG-based sensors, optical fiber, a static fiber grating demodulation instrument (SM125), and a PC with the necessary software. Experimental data was collected in the presence of an artificial landslide, and the results show that the lower FBGs are more sensitive than the upper FBGs for all four of the custom sensors. It was also found that Sensor 2 and Sensor 4 are more capable of monitoring small-scale landslides than Sensor 1 and Sensor 3, and this is mainly due to their placement location with respect to the landslide. The stronger rods used in the hard sensors make them more adaptable to the harsh environments of large landslides. Thus, hard sensors should be fixed near the landslide, while soft sensors should be placed farther away from the landslide. In addition, a clear tendency of strain variation can be detected by the soft sensors, which can be used to predict landslides and raise a hazard alarm.

## 1. Introduction

Landslides (also known as landslips) are one of the most costly geological hazards, and they can occur in many regions all around the world [1]. The mechanism that causes landslides is extremely complicated, making it difficult to monitor a landslide or predict its location and time of occurrence [2]. However, the large potential for loss of life and property that are associated with landslides has made their study an important area of research. Scientists around the world have proposed various methods of monitoring the position of landslides, and these fall into two main types: one is displacement monitoring at the surface level, and the other is internal strain/displacement monitoring of the slope body.

In recent years, displacement-based sensors with high precision have been used in the method of monitoring the surface of landslides. Global Navigation Satellite System (GNSS) techniques have also been used to measure the changes of the three-dimensional positions on the surface of the slope. These changes include rapid and continuous static surveys in scattered single monitoring points or baseline vector modes [3,4,5,6,7,8,9,10]. Although GNSS-based methods can provide millimeter positioning accuracy and continuous monitoring, only a limited number of monitoring points can be used, which may not completely cover the region of the slope body. In contrast, remote sensing techniques can obtain massive amounts of point position information of the slope with high accuracy, e.g., InSAR (Interferometric Synthetic Aperture Radar) and LIDAR (Light Detection and Ranging). However, both of these techniques have poor real-time performance in deformation monitoring [11,12,13,14,15,16]. In other words, the general method of displacement monitoring has difficulties when trying to cope with the internal changes of the slope body, which can present a barrier in the monitoring and prediction of landslides.

The second type of method is based on internal strain/displacement monitoring, which can probe into the slope body. This enables the collection of strain, displacement, and stability parameters. Traditional implementations of this method include electric-based geotechnical instruments and sensors, which are now being gradually replaced with optical-based sensors. Novel optical-based sensors have been proposed in recent years, such as the fiber Bragg grating (FBG)-based stress monitoring sensor developed for riser safety monitoring [17]. Pei et al. [18] presented a FBG-based inclinometer for slope monitoring, which was capable of measuring the lateral movement of the slope. Ho et al. [19] developed a FBG-based deflectometer for ground movement monitoring. Maneesha et al. [20] presented a wireless sensor network consisting of deep-earth probes that used FBG strain gauges to monitor landslides in India. Landslides and debris flows were monitored and predicted by a FBG-based inclinometer and a FBG-based column-net system in the Weijiagou Valley of China [21]. A slow-moving landslide was monitored by BOTDR (Brillouin optical time domain reflectometry), however, this technique has the disadvantage of poor real-time performance [22]. Embedded OTDR sensors have also been used as distributed strain sensors for long distances in landslide monitoring [23]. All of the above research was based on actual landslides, and some had difficulty monitoring the landslide phenomena in real-time.

In this paper, an artificial hillock and landslide is simulated and monitored by custom FBG-based sensors. Two types of FBG-based sensors are proposed and designed for this experiment, and the performance of these sensors is assessed in a series of artificial landslides. Compared with the existing research, this study presents an alternative package design and packaging process for the sensors, and uses an artificial landslide as the test method. The artificial landslide used in the experiments presented here can be controlled, while the experiments in references [18,19,24] depended either on natural landslides or simply on the deformation of soil. In terms of the sensor design, the current references [18,24,25] used either rigid plastic or steel rods; in the experiment present here both types of rods are used to enable an accurate characterization and comparison. In addition, a special multi-layer packaging technology was used to ensure a higher level of reliability or the sensors in complex environments.

This paper is organized as follows: Section 2 briefly describes the principle of FBG sensing technology; Section 3 details the construction of the FBG-based sensors, the design, and packaging method used for the FBG sensors, and the sensor calibration; the experimental setup and the analysis of the resulting sensor data when monitoring the artificial landslide are presented in Section 4; and finally, conclusions and deductions are given in Section 5.

## 2. Principle of FBG Sensing Technology

FBG sensors offer clear advantages over electrical sensors due to their use of light rather than electricity. Strain and temperature can be measured by FBG sensors over long distances with little or no loss in performance or signal integrity. In addition, the copper wire used by electric sensors is replaced by optical fiber for the optical sensors; since the FBG sensors and associated optical fiber are not conductive, this type of system does not suffer from noise due to electromagnetic interference. Another difference when compared to electrical sensors is that each optical channel can link a number of fiber sensors, which allows FBG sensing systems to have the advantages of small size, light weight, and low complexity. The construction and sensing principle of an FBG will be described next.

**Figure 1:**
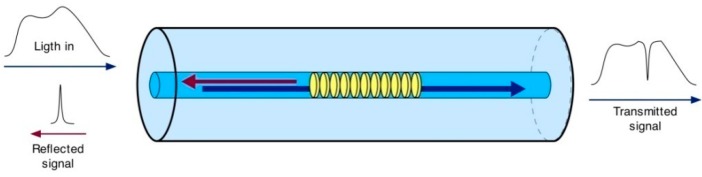
Working mechanism of a FBG sensor.

To fabricate a FBG, a periodic variation in the refractive index of a fiber is created by exposing it to light of an appropriate intensity. This creates a structure that reflects a specific wavelength of light, and this reflected wavelength shifts in response to changes in temperature and strain. It is this property that is exploited to realize a sensor. The working mechanism of a FBG sensor is depicted in Figure 1.

When a broad spectrum of light is transmitted to the Bragg grating, only a specific wavelength of light is reflected. This is known as the Bragg wavelength, which can be expressed as

(1) $$\lambda_{B}=2n_{eff}\Lambda$$

where *n_eff_* is the effective refractive index of the fiber core, and Λ is the period of the Bragg grating, which is the spacing between the gratings.

The Bragg wavelength (i.e., the central wavelength of the reflected signal) is influenced by *n_eff_* and Λ. A shift in the reflected wavelength is caused by a change in the strain and/or temperature in the external environment. As a result, the relationship between the reflected wavelength and the measured parameters can be established. The relationship between the shift in the reflected wavelength ∆*λ*_B_ and the change in strain ∆*ε* is given by [26,27]

(2) $$\frac{\Delta \lambda_{B}}{\lambda_{B}}=(1-P_{e})\Delta \varepsilon +(a_{\Lambda }+a_{n})\Delta T$$

where *P_e_* is the strain-optic coefficient, ∆*λ_B_* and *λ_B_* are the shifted and initial wavelengths, respectively, *a*_Λ_ is the coefficient of thermal expansion, *a_n_* is the coefficient of temperature sensitivity, ∆*ε* is the change in the strain, and ∆*T* represents the change in the temperature.

Since the reflected wavelength of a FBG is affected by both temperature and strain, it is necessary to compensate for the influence of temperature on the FBG output in actual measurement applications.

## 3. Design and Calibration of FBG-Based Sensors

### 3.1. Design and Packaging Technology of FBG-Based Sensors

**Figure 2:**
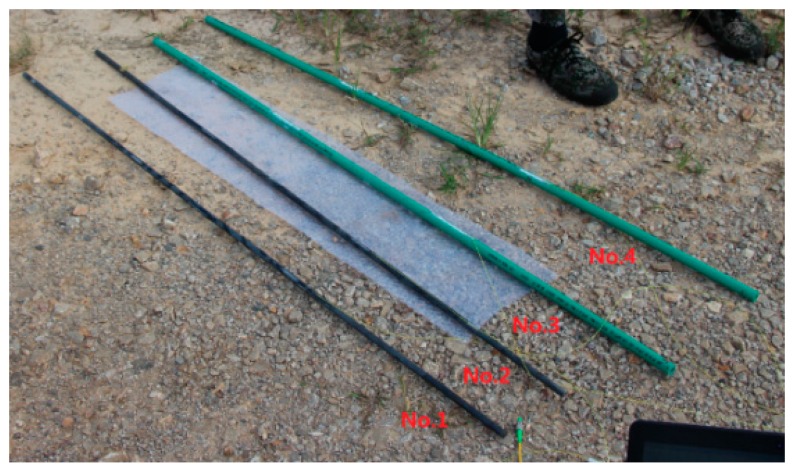
The four sensors used in this study (bare sensors).

Four custom FBG-based sensors were designed and implemented for the landslide monitoring experiment in this study, and these are shown in Figure 2. Sensor 1 and Sensor 2 are based on a high-strength reinforced steel bar, while Sensor 3 and Sensor 4 are based on a rigid plastic bar with low strength. In this paper, Sensor 1 and Sensor 2 are referred to as “hard sensors” and Sensor 3 and Sensor 4 are referred to as “soft sensors”. The strength and Young’s modulus of the steel rods (hard sensors) are 335 MPa and 200 GPa, respectively, while the strength and Young’s modulus of the rigid plastic rods (soft sensors) are 0.4 MPa and 3.8 GPa, respectively. All of the sensors have a length of 150 cm and a diameter of 0.8 cm. Special packaging technology was used to fix two FBGs to each sensor rod at distances of 50 cm and 100 cm from the top of the rod. The central wavelengths of the two FBGs are 1525 nm and 1520 nm, respectively.

**Figure 3:**
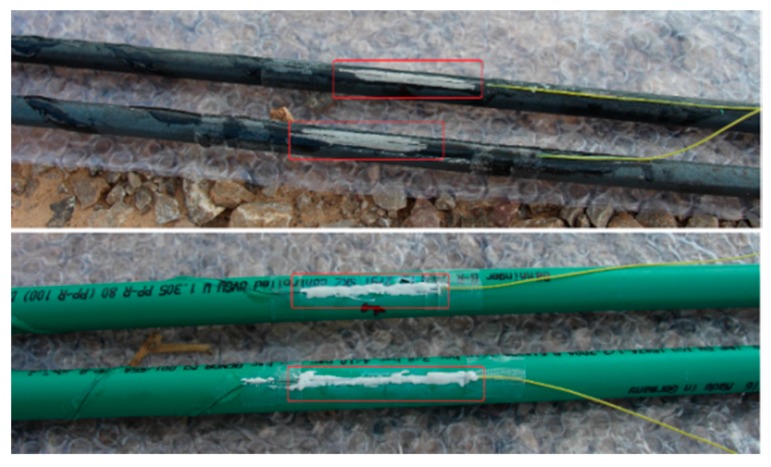
Protective epoxy resin layer for bare fiber.

**Figure 4:**
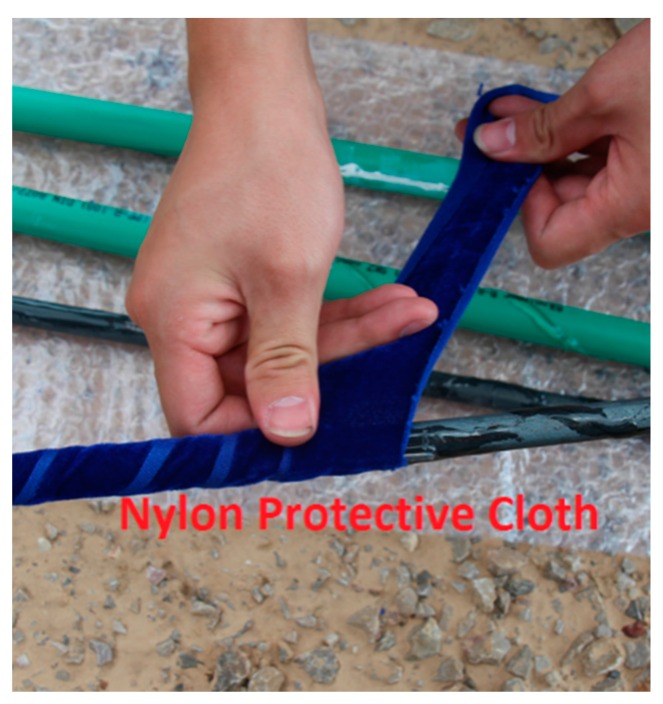
Protective nylon cloth layer.

**Figure 5:**
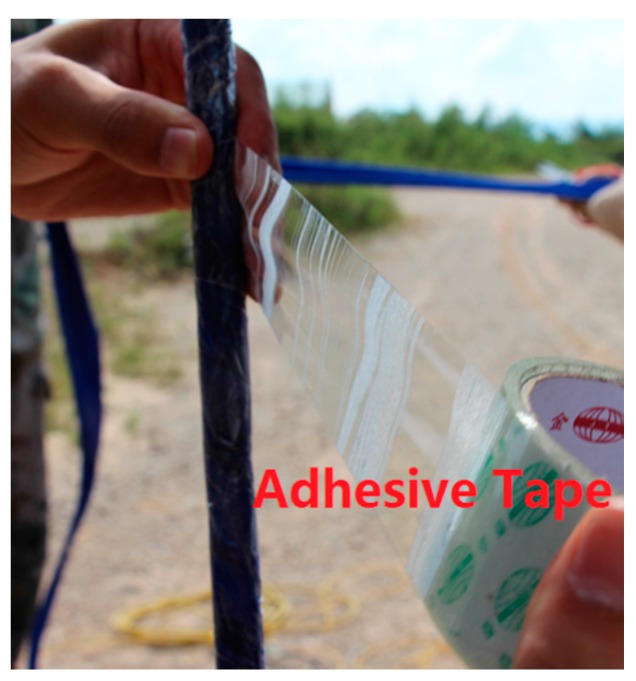
Protective adhesive tape layer.

The packaging technology used in these sensors is complicated, but it can be simplified into the following process:

- **Step1:** Loctite 401 multi-purpose super strong instant adhesive glue is used to fix two fiber gratings onto the rod (steel/plastic) of a sensor.
- **Step2:** In order to prevent the sensors from being damaged during the experiment, any bare fiber is coated with epoxy resin as shown in Figure 3.
- **Step3:** After the solidification of the epoxy resin, nylon protective cloth is used to protect the rod of the sensor by using the winding reinforcement scheme shown in Figure 4.
- **Step4:** Lastly, adhesive tape is used to ensure the smoothness of each rod, as shown in Figure 5.

### 3.2. Sensor Calibration

**Figure 6:**
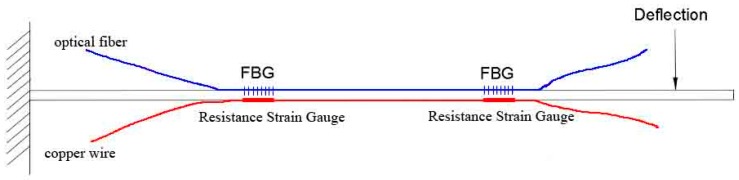
Sensor calibration setup.

To calibrate the sensors, each FBG was paired with a resistance strain gauge and both devices were mounted onto a steel bar so the readings could be compared. All of the sensors were affixed at distances of 50 cm and 100 cm from the top of rod, as shown in Figure 6.

**Figure 7:**
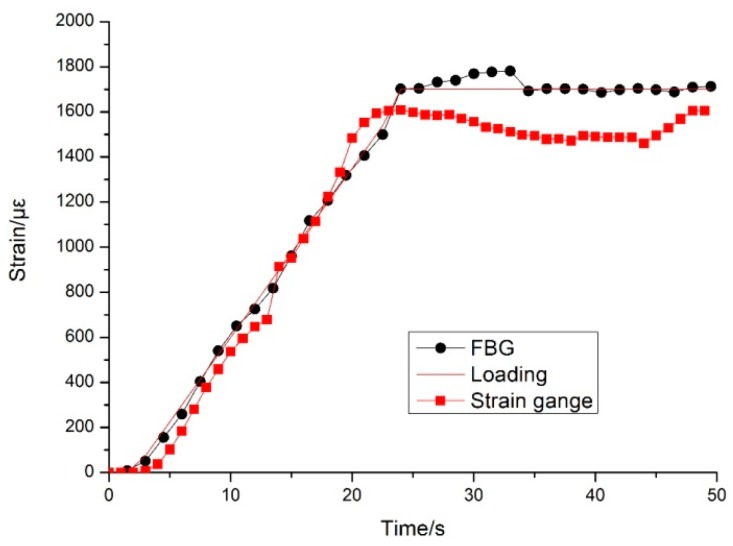
Strain measured by the FBG sensor and the strain gauge during the loading process.

Two experiments were implemented to calibrate the FBG-based sensors. The first experiment was to compare the measurement accuracy between the FBG and the resistance strain gauge. In the process of unilateral loading, the FBGs were subjected to tensile force, while the resistance strain gauges were placed under pressure. Both the tensile force and the pressure force should be theoretically consistent with each other. The experimental results plotted in Figure 7 show that the strain of the FBG and the resistance strain gauge are linearly related to the loading stress. The horizontal axis shows the loading time, and the vertical axis shows the strain. The theoretical loading, FBG output, and strain gauge output are all shown in Figure 7, and we can see that the FBG has a higher accuracy and stability than the resistance strain gauge. 

**Figure 8:**
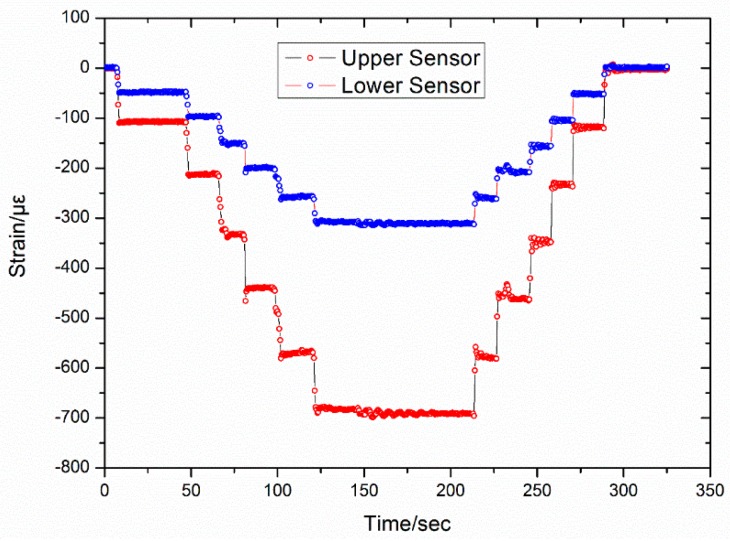
Comparison between the upper and lower FBG.

The second experiment was a comparison of the strain measurement between the two FBGs on each rod, which are located at distances of 50 cm and 100 cm with respect to the top of the rod. The sensor was fixed and installed on a Mechanical Testing & Simulation (MTS) test machine, and a migration sensor was fixed on the inter-locking nail. Accurate deformations of the loading point were carried out in 1 cm increments. A 6 cm descending displacement was performed first, after which a 6 cm ascending displacement was performed. In both cases, the displacement step size was 1 cm. The results in Figure 8 show that when a 1 cm displacement was applied to the top of the rod, nearly 100 με and 50 με of strain was on the FBG sensors at 50 cm and 100 cm, respectively.

## 4. Experiment Results of Artificial Landslides Monitoring

**Figure 9:**
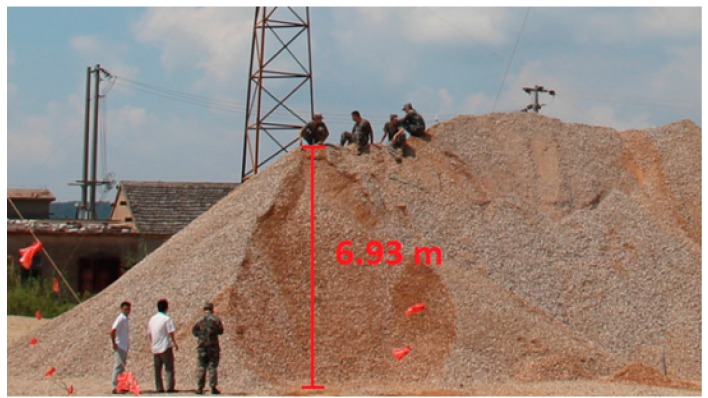
Artificial hillock.

The landslide monitoring experiment setup consists of a 6.93 m high hillock of piled up stones and sand in Tangshan, a village located in Nanjing, Eastern China. This hillock is shown in Figure 9, and the four custom sensors and corresponding instruments were used to collect data on the landslide.

The geo-materials of the hillock were mainly gravel bluestone from Jiangsu, China. Their particle size is 5–30 mm, and their elastic modulus is 10,373 MPa. The dry density and the friction angle is 1.8 g/cm^3^ and 44.5°, respectively. The particle size distribution is shown in Table 1.

**Figure 10:**
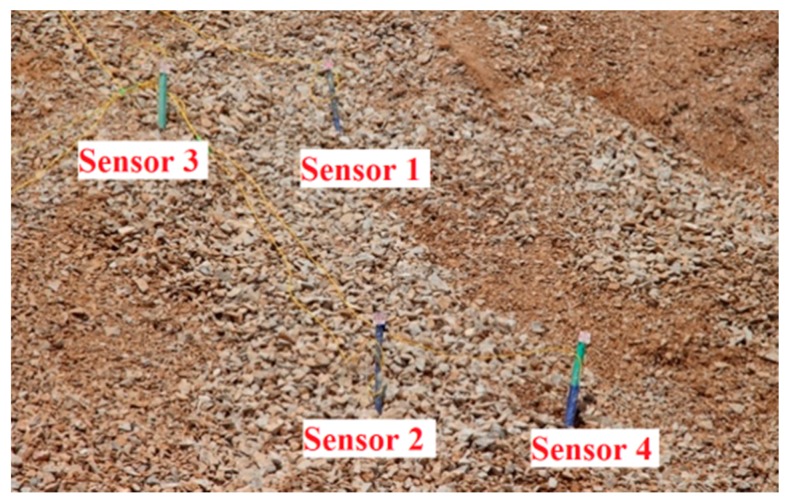
Location of the four sensors.

**Figure 11:**
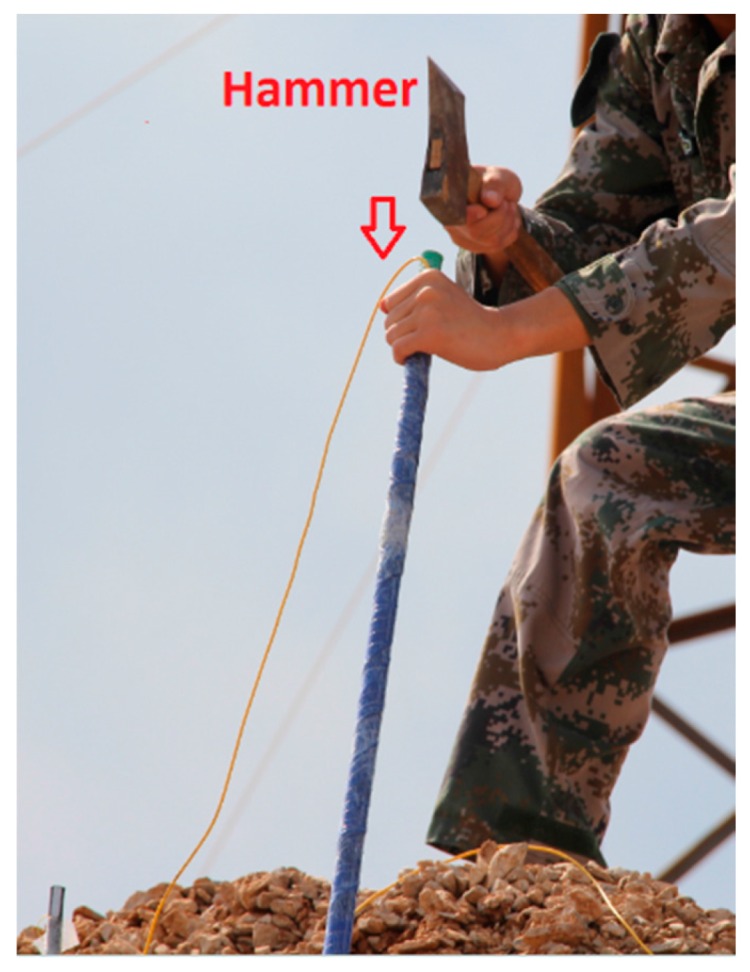
Method of installing the sensors.

In this experiment, surface deformation and displacement are the intuitive phenomena of the slope destruction, while the internal strain variations are the intrinsic cause. As shown in Figure 10, the four custom sensors were installed at four selected locations. There are two FBGs on each sensor, and the sensors were installed into the prepared boreholes. The bottom of the sensors was deeply buried in the internal stability zone of the slope. The sensor was knocked into the soil with a hammer, as shown in Figure 11.

**Figure 12:**
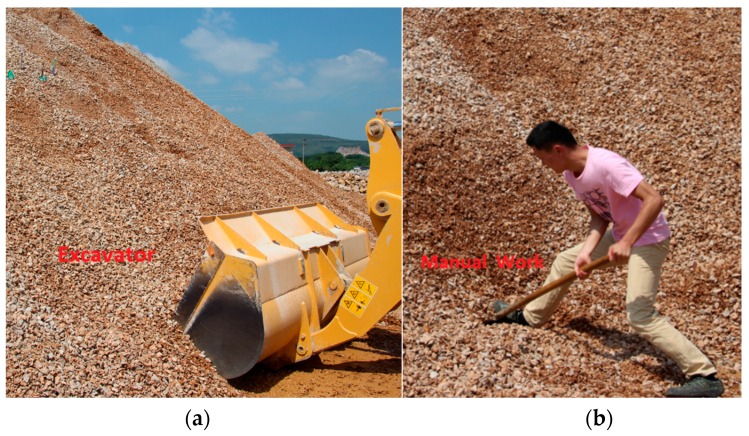
(**a**) Digging out the slope with an excavator; (**b**) Manually digging out the slope.

Digging into the lower part of the conventionally stable slope was used to initiate the process of a landslide. By digging into the lower part of the slope, the upper part of the slope loses stability due to the lack of carrying capacity, and this causes the artificial landslide to occur. To accomplish this process, an excavator was first used to carry out a large volume of stones and sand, as shown in Figure 12a. When the slope started to become structurally loose, the excavator was replaced with manual digging as shown in Figure 12b. This excavation method results in a slower landslide, which allows more data to be recorded by the FBG-based sensors.

**Figure 13:**
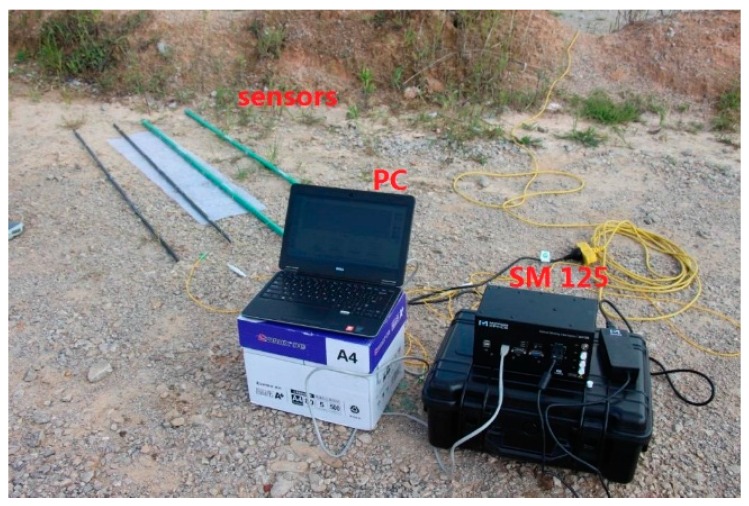
The sensing system incorporating the custom sensors.

The FBG-based strain monitoring system was developed to meet the needs of landslide monitoring, and consists of four FBG-based sensors, a fiber grating demodulation instrument, and a computer and software (as shown in Figure 13). The FBGs and static fiber grating demodulator are made by Micron Optics, Inc., Atlanta, GA, USA and their model numbers are SMF-28C and SM125, respectively.

**Figure 14:**
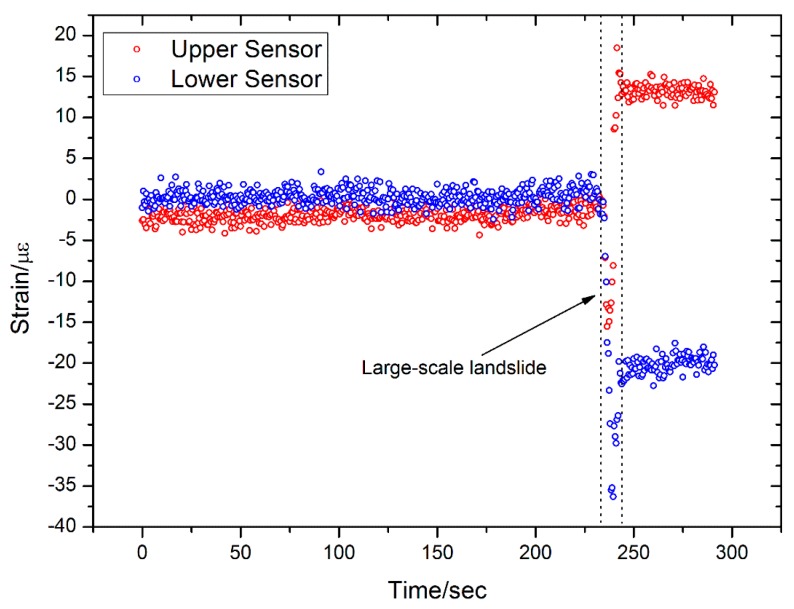
Results from Sensor 1.

**Figure 15:**
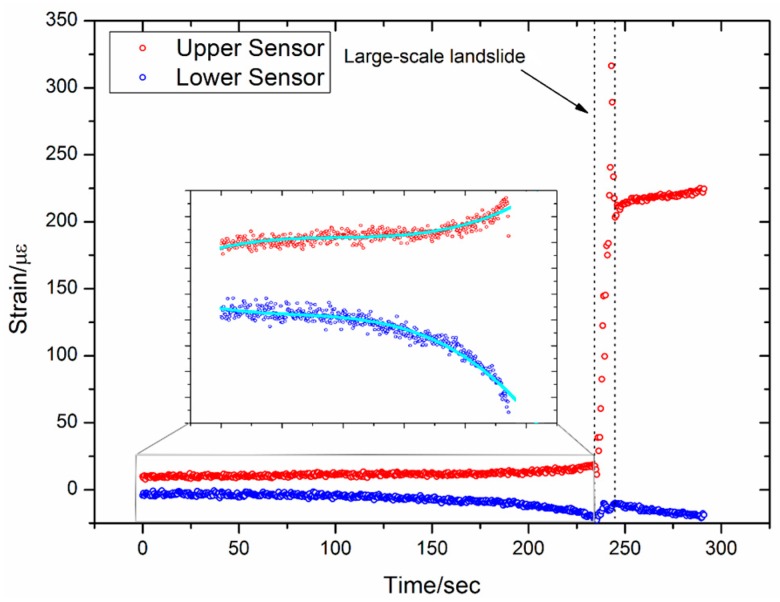
Results from Sensor 3.

**Figure 16:**
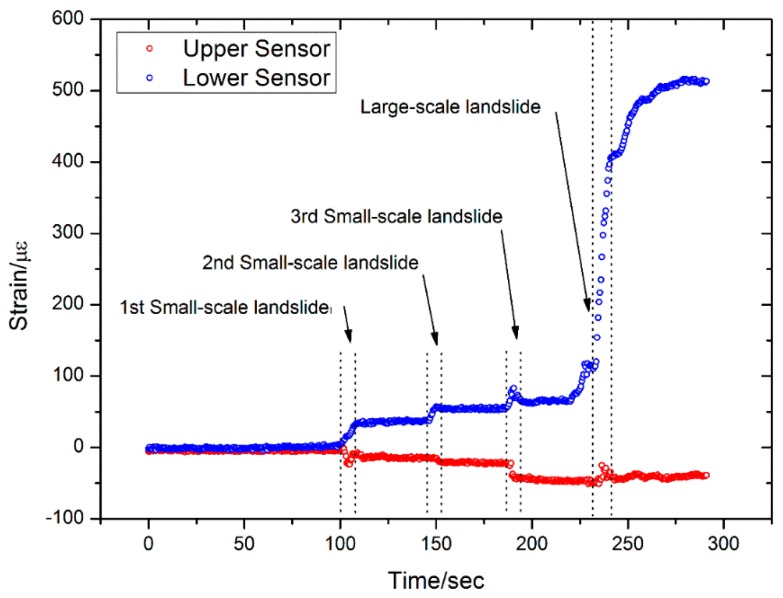
Results from Sensor 2.

**Figure 17:**
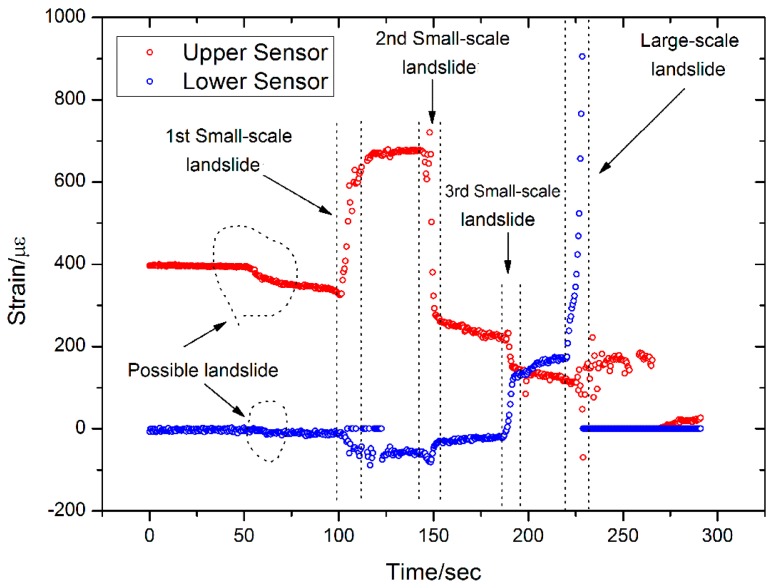
Results from Sensor 4.

The following sequence occurs during the creation of the artificial landslide: the slope is gradually loosened by the excavator digging, which results in some small-scale landslides. At an elapsed time of 240 s, a large-scale landslide occurs and causes serious structural damage to the hillock. The placement of the four sensors leads to differences in their associated strain data. Sensors 2 and 4 are near the landslide body, while Sensors 1 and 3 are some distance away from the landslide body. The data collected by the four sensors are shown in Figure 14, Figure 15, Figure 16 and Figure 17. The sizes of the landslides in this paper are mainly defined by the visual observations of the authors. A small-scale landslide corresponds to less than one square meter of landslide in surface area, while a large-scale landslide corresponds to more than one square meter of landslide in surface area.

The results from Sensor 1 are shown in Figure 14. We can see that there is little strain variation in either of the FBGs that are at 50 cm and 100 cm from the top of the sensor before the large-scale landslide. However, when the large-scale landslide occurs, both FBGs record a significant “jump” in the strain data. The FBG that is 50 cm from the top measures 15 µε, while the FBG that is 100 cm from the top measures −20 µε. The latter sensor is, therefore, more sensitive in monitoring the landslide.

The results from Sensor 3 are shown in Figure 15. This sensor is at almost the same height as Sensor 1, and is subjected to similar stress and strain. However, Sensor 3 is a soft sensor, whereas Sensor 1 is a hard sensor. As shown in Figure 15, the flexibility of this soft sensor allows Sensor 3 to monitor both the large-scale landslide and also the trend of the strain variation during the small-scale landslides. This is especially noticeable in the lower FBG (100 cm from the top), and this type of data is crucial for landslide prediction. 

Sensor 2 is a hard sensor, and is located near the landslide body. Three small-scale landslides and a large-scale landslide were recorded by the sensor in this experiment. Figure 16 shows that a data “jump” is generated at each landslide, which is can be used as a landslide indicator. The lower FBG is observed to be more sensitive than the upper FBG, as evidenced by the bigger jump.

**Figure 18:**
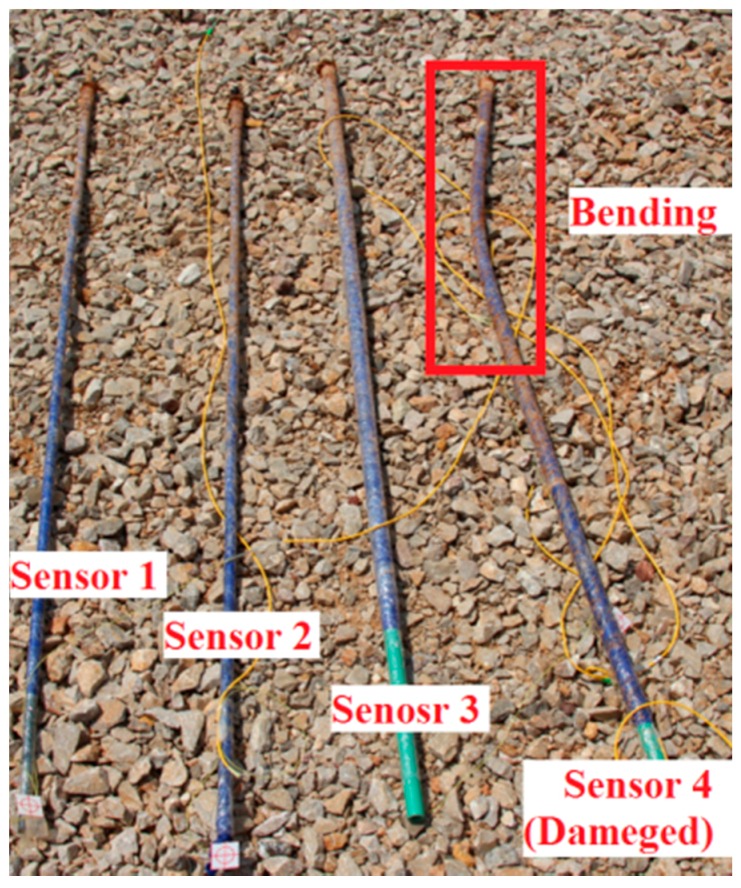
Sensors after the artificial landslide.

The results from Sensor 4 are shown in Figure 17, and it is at almost the same height as Sensor 2 and is subjected to similar stress and strain. The main difference is that Sensor 4 is a soft sensor, whereas Sensor 2 is a hard sensor. Five landslides can be observed in the monitoring results from Sensor 4, four of which are similar to the results from Sensor 2. However, a possible landslide was detected by the upper FBG at an elapsed time of 100 s. During the last large-scale landslide, the lower FBG on Sensor 4 was damaged and all sensor data was erased. A picture of the sensors after the artificial landslide is shown in Figure 18, and the damage to Sensor 4 is clearly visible.

Table 2 summarizes the data collected during the experiment of FBG-based sensors in landslide monitoring.

The following conclusions can be drawn from the experiment:

(1) Better results are obtained when monitoring small-scale landslides near the landslide body, such as with Sensors 2 and 4. In contrast, the sensors those are located some distance away from the landslide body can only detect the large-scale landslide. Several possible small-scale and large-scale landslides have been measured with Sensors 2 and 4.
(2) Each sensor has two FBGs—an upper FBG and a lower FBG—and the lower FBG is more sensitive to the small-scale landslides. This can be directly observed from Figure 14 and Figure 16, which show that the variations of the lower FBG outputs are larger (more obvious) than the upper FBG outputs.
(3) Figure 17 and Figure 18 show the results obtained from a large-scale landslide, and it was found that the soft sensor can be easily damaged when it is close to the landslide, while the hard sensor is more durable and can withstand the associated force without damage.
(4) When the sensor is located far away from the landslide body, small-scale landslides are almost impossible to detect with hard sensors. However, the soft sensors can be used as comparative devices as they can detect a varying trend of strain in the landslide body, which is very critical for early landslide warnings.

## 5. Conclusions 

Fiber optical sensors show a clear advantage in obtaining accurate strain information on landslides because they are not affected by electromagnetic interference. FBG-based sensors have been recognized as a promising technology for continuous monitoring of landslides.

In this work, a set of custom FBG-based sensors were installed in boreholes on the landslide body to monitor an artificial landslide. Results of the experiment show that the sensors exhibit good accuracy and sensitivity for this application and two main conclusions can be drawn. First, hard sensors should be installed closer to the landslide body (as opposed to soft sensors) because they can withstand the larger stress that is present in this area. Second, there should be a certain distance between soft sensors and the landslide body, and the mechanisms of inner deformation in the slope can be obtained by analyzing the tendency of the strain data collected by these sensors. Despite the significant advantages mentioned above, the cost of FBG-based sensors remains an issue. The price of an FBG-based sensor is about $20, while the strain gauge resistance-based sensor is about $5. FBG sensors are still more expensive than electric sensors at present, but with the development of optical sensing technology, the price may fall in the future.

The sensor design method and the sensing system presented here is useful for monitoring the kinematics and evolution of landslides in real-time. Future research will focus on developing methods to predict landslides using tendency data such that a hazard alarm system can be developed.

## Figures and Tables

**Table 1:** The particle-size gradation of the crushed gravel soil.

**Radius/mm**	5–10	10–15	15–20	20–30
**Mass Percent/%**	8.2	20.4	31.9	39.5

**Table 2:** Monitoring results of different sensors.

Landslide Type		Far from the Landslide Body		Near the Landslide Body	
		Sensor 1	Sensor 3	Sensor 2	Sensor 4
**Possible**	1	None	None	None	Detectable
**Small-Scale**	2	None	A trend of destruction	Detectable	Detectable
	3	None		Detectable	Detectable
	4	None		Detectable	Detectable
**Large-Scale**	5	Detectable	Detectable	Detectable	Detectable
